# Time-Lapse Imaging as a Tool to Investigate Contractility of the Epididymal Duct – Effects of Cgmp Signaling

**DOI:** 10.1371/journal.pone.0092603

**Published:** 2014-03-24

**Authors:** Andrea Mietens, Sabine Tasch, Angelika Stammler, Lutz Konrad, Caroline Feuerstacke, Ralf Middendorff

**Affiliations:** 1 Institute of Anatomy and Cell Biology, Justus-Liebig-University Giessen, Giessen, Germany; 2 Department of Gynecology and Obstetrics, Justus-Liebig-University Giessen, Giessen, Germany; University of Debrecen, Hungary

## Abstract

The well orchestrated function of epididymal smooth muscle cells ensures transit of spermatozoa through the epididymal duct during which spermatozoa acquire motility and fertilizing capacity. Relaxation of smooth muscle cells is mediated by cGMP signaling and components of this pathway are found within the male reproductive tract. Whereas contractile function of caudal parts of the rat epididymal duct can be examined in organ bath studies, caput and corpus regions are fragile and make it difficult to mount them in an organ bath. We developed an *ex vivo* time-lapse imaging-based approach to investigate the contractile pattern in these parts of the epididymal duct. Collagen-embedding allowed immobilization without impeding contractility or diffusion of drugs towards the duct and therefore facilitated subsequent movie analyses. The contractile pattern was made visible by placing virtual sections through the acquired image stack to track wall movements over time. By this, simultaneous evaluation of contractile activity at different positions of the observed duct segment was possible. With each contraction translating into a spike, drug-induced alterations in contraction frequency could be assessed easily. Peristaltic contractions were also detectable and throughout all regions in the proximal epididymis we found regular spontaneous contractile activity that elicited movement of intraluminal contents. Stimulating cGMP production by natriuretic peptide ANP or inhibiting degradation of cGMP by the phosphodiesterase 5 inhibitor sildenafil significantly reduced contractile frequency in isolated duct segments from caput and corpus. RT-PCR analysis after laser-capture microdissection localized the corresponding molecules to the smooth muscle layer of the duct. Our time-lapse imaging approach proved to be feasible to assess contractile function in all regions of the epididymal duct under near physiological conditions and provides a tool to evaluate acute (side) effects of drugs and to investigate various signaling pathways.

## Introduction

Epididymal function supports male fertility by ensuring maturation of spermatozoa during their transit through the organ. When released from the seminiferous epithelium in the testis, spermatozoa are still immotile and unable to fertilize an egg. They have to transit through the efferent ducts which connect the rete testis with the epididymal duct, and then travel along the further parts of caput, the corpus and the cauda epididymidis. During their transit through the epididymis, spermatozoa undergo further maturation and acquire motility [1], [2]. Exposure of spermatozoa to varying microenvironments along the tubular system of the epididymis (efferent ducts, initial segment, further parts of the epididymal duct) is believed to be crucial for appropriate maturation [3]. Within the distal cauda epididymidis, spermatozoa are stored until ejaculation. Here, transport of spermatozoa is mainly controlled by neuronal activity [4], [5]. However, passage of spermatozoa through the entire rat epididymal duct takes up to 8 days [6] and needs to be well orchestrated while innervation in the caput and corpus region of the organ is scarce [4], [7]. Accelerated transit time has been associated with reduced fertility [6] underlining the importance of regulating the transport of immotile spermatozoa within the epididymis. Net fluid flux resulting from epithelial secretions may contribute to the transport of spermatozoa, but transportation mainly relies on the contractile activity of the smooth muscle cell layer surrounding the epididymal epithelium [3]. This contractile activity is mostly regulated by paracrine and endocrine factors [6], [8], [9]. Various signaling systems known to alter smooth muscle activity have been localized to the epididymis and can therefore affect epididymal function and male fertility. Among factors enhancing epididymal smooth muscle contraction are oxytocin, endothelin or noradrenaline [8], [10]–[12]. Since most investigations about contractile function focus on the vas deferens or its epididymal beginning, there is little functional data on the epididymal duct itself and especially its proximal parts.

Smooth muscle relaxation is known to be elicited by cGMP-related signaling [13]. Previously, the occurrence of components of the cGMP pathway within the epididymal smooth muscle layer has been demonstrated and contractility of the duct was shown to be diminished by cGMP in the mid-cauda region by organ bath studies [14]–[16]. Functional data on more proximal regions of the duct (corpus and caput) are limited in rodents. Since epididymal smooth muscle function and its regulation is an important factor contributing to male fertility, we sought to establish a procedure that allows to assess contractility of proximal parts of the epididymal duct in a near physiological setting. Notably, many drugs that are used therapeutically modulate epididymal or vas deferens smooth muscle function as for example anti-hypertensive drugs [17], [18]. However, very little is known about potential effects or side effects on extra-vascular smooth muscle cells related to epididymal function. Here, we present a reasonable approach to investigate epididymal smooth muscle function in a near-physiological setting. Our time-lapse imaging procedure allowed us to appreciate spontaneous contractile activity in the epididymal duct, to assess the pattern, frequency and propagation of contractions as well as drug-induced alterations. In contrast to experiments using isolated and cultured cells, our approach respects the integrity of the epididymal duct segments. This approach may provide a useful tool to study epididymal contractile function *ex vivo* and how it is affected by various drugs or pathological conditions, e.g. infection or inflammation.

## Materials and Methods

### Ethics statement

Epididymal tissues were obtained from adult Wistar rats housed in the animal facility of Justus-Liebig-University Giessen. Housing, animal care and all procedures were conducted according to the guidelines for animal care and approved by the committee for laboratory animals of Justus-Liebig-University Giessen, case number JLU-Nr.419_M.

### Material

Rats were anesthetized with 5% isoflurane prior to sacrifice by cervical dislocation. Epididymal tissue was removed and and either immediately frozen in liquid nitrogen for laser-assisted capture microdissection or kept in minimal essential medium (MEM; Gibco, Invitrogen, Karlsruhe, Germany) at 4°C until immediate preparation within the next one to two hours. Parts of the epididymal duct were isolated by careful dissection under direct observation with a binocular microscope. Parts of up to 10 to 20 mm length were cut from caput (segments 3 to 8, mainly segments 5 and 7, according to Turner et al. [19]) and corpus (segment 12) regions and further prepared for time-lapse imaging as described below. The initial segment (IS), considered functionally and morphologically different from the other parts of the caput epididymidis and to be a site of intense fluid resorption [20], was not included in our study.

### Laser Capture Microdissection (LCM) and RT-PCR

A PALM LCM System with autocatapult and Robocut Software (Zeiss, Munich, Germany) was used for microdissection. Frozen specimens of rat epididymis were cut (10 μm) and serial sections were transferred to PALM Membrane Slides (Zeiss). Cryosections were immediately stained with hematoxyline and fixed with ethanol. Approximately 10 min before microdissection, individual slides were air-dried at room temperature. Within epididymal duct tissue, samples from the smooth muscle layer were microdissected and catapulted into sterile oil covered cups of 0.5 ml. Every single cup used for repeated experiments contained the same extent of respective tissue area (500,000 μm^2^). For RNA isolation, the tissue samples were dissolved in RLT-buffer (Qiagen, Hilden, Germany) with β-mercaptoethanol and snap-frozen on dry ice. Total RNA was extracted using the Qiagen RNeasy micro kit with inclusive DNase on column digestion. Eluted RNA was used as a template for the synthesis of first strand cDNA using reverse transcriptase (iScript cDNA synthesis kit, Biorad, Munich, Germany), followed by PCR amplification (AmpliTaq Gold kit, Applied Biosystems, Life Technologies, Carlsbad, CA, USA). Whole epididymis tissue preparations served as positive control. The following intron spanning primers were used for amplification: GC-A-forward (5′-CTGAGGCCTTGCTTTACCAG-3′) and GC-A-reverse (5′-TTGAGCAGAGTCACCACCTG-3′) resulted in a 172 bp fragment (NM_012613). The sequence for GC-B (NM_053838.1) contained forward (5′-AATGTCGTTGCCATCAAACA-3′) and reverse (5′-GAGCGAGTAGCGAAACATCC-3′) primers with a fragment size of 234 bp. The forward primer for sGC (NM_012770.2) was (5′- GCCAATGAGCTGAGACACAA-3′) and the sequence for the reverse primer was (5′- ACAAGGTTCTGGCAAACCAC-3′). As positive control for smooth muscle cells, we used smooth muscle actin (SMA), (NM_031004) forward primer (5′- TGTGCTGGACTCTGGAGATG-3′) and reverse primer (5′- GAAGGAATAGCCACGCTCAG -3′), amplifying a 148 bp fragment. For amplification of the 259 bp fragment of transient receptor potential cation channel V6 (TRPV6) (NM_053686.1)] as positive control for the epithelial layer [21] we used following oligonucleotides: TRPV6-forward (5′-CTGCAAGTCCAAGGACAAGG-3′) and TRPV6 reverse (5′-GGTCCTCTGAAGCACTCTGG-3′). For amplification of the 292 bp sized housekeeping gene RPS18, forward primer (5′- GTGATCCCCGAGAAGTTTCA -3′) and reverse primer (5′- TGGCCAGAACCTGGCTATAC -3′) were used (NM_213557.1). The samples were denaturated at 95°C for 12 min, followed by 45 cycles of each 30 s at 95°C, 20 s at 59°C and 30 s at 73°C. Finally, PCR samples were kept at 73°C for 7 min and stored at 4°C. Amplified products were separated by electrophoresis on 2% agarose gels.

### Preparation of epididymal duct segments for time-lapse imaging

Duct segments were placed in a microscopy well straightened and fixed between fine needles at their extremities, but otherwise floating in medium. Alternatively, collagen embedding (see below) was used as a means to immobilize the duct segments.

### Preparation of collagen stock solution and collagen embedding of epididymal duct segments

A collagen stock solution was prepared using collagen fibres isolated from four rat tails conserved at −20°C. Rat tails were left to thaw in 70% ethanol and denuded. Collagen fibres were pulled from the tissue and soaked in 70% ethanol for 15 to 20 minutes. After drying, fibres were agitated at 4°C in 250 ml 0.1% acetic acid for 48 hours. The resulting viscous collagen suspension was centrifuged at 4°C at 24,000 g under sterile conditions. The clear supernatant was stored at 4°C.

One gram of collagen was mixed with 125 μl of 10×DMEM/F12 (Gibco, Invitrogen, Karlsruhe, Germany), 30 μl glacial acetic acid, 42 μl 0.5 M NaOH and 22.5 μl HEPES with vortexing after adding every substance. Upon addition of NaOH, the solution turns from pale yellow to a pink colour and back to yellow when HEPES is added. Collagen started to polymerize promptly and the liquid collagen was immediately placed at the bottom of the microscopy well. The epididymal duct segment was placed in the middle and care was taken to cover the duct segment with the collagen to prevent it from drying. After polymerization at 37°C in an incubator, the wells were covered with 1 ml of medium.

### Time-lapse imaging

For time-lapse imaging, the wells containing the isolated duct segments were kept at a temperature of 34°C. Picture frames were typically taken at an interval of 1 s. Additionally, imaging was accelerated to 7 frames/s to allow following the wall movements with a finer time resolution.

We monitored the duct segment under transmitted light with a magnification of 100× or 200× and used an Olympus BX50WI microscope equipped with UMPlan Fl 10×/0,5 W and UMPlanFl 20×/0,5 W objectives, and a Till-Imago QE CCD camera (Photonics, Gräfelfing, Germany) combined with TillVision software (Version 4.0, Photonics). Further analysis of the movies was done using ImageJ 1.43u (public domain software, NIH, USA, download at http://rsb.info.nih.gov/ij).

We observed spontaneous contractile activity in caput and corpus and focused our investigation on these regions. Rarely, no spontaneous contractile activity occurred. Since accidental injury to the epididymal duct during preparation that would not be discernible by transillumination microscopy was always a concern, we considered spontaneous contractile activity a proof of integrity of the tissue and disregarded duct segments that did not show spontaneous contractility.

To visualize contractile activity, time-lapse images were treated as a stack of picture frames taken at a regular time interval (time stack). By placing a virtual section through the wall of the epididymal duct, this particular part of the duct could be followed over time through the time stack. Each contraction corresponds to a movement of the wall which was translated into a small peak when followed over time. This visualization allowed to count the contractions within a defined period to assess frequency, but also provides a quick overview on the contractile pattern.

### Investigation of drug effects and signaling pathway components

To test whether spontaneous contractile activity was susceptible to cGMP-related effects, different substances were used. The natriuretic peptides atrial natriuretic peptide (ANP) and C-type natriuretic peptide (CNP) enhance cGMP generation by stimulating the particulate guanylyl cyclases A (GC-A) and B (GC-B), respectively. The nitric oxide donor sodium nitroprusside (SNP) stimulates soluble guanylyl cyclase (sGC) to generate cGMP whereas sildenafil, a specific PDE5 inhibitor, increases cGMP levels by inhibiting its degradation by phosphodiesterase 5 (PDE5). ANP and CNP (both from Bachem, Weil am Rhein, Germany) were used at a final concentration of 0.1 μM, SNP (sodium nitroprusside, Fluka 71780, Buchs, Switzerland) was used at 100 μM and sildenafil (Pfizer, Karlsruhe, Germany) at 5 μM. Concentrations of the tested substances were used according to previous experiments [15], [22], [23].

### Statistical analyses

To evaluate the effects of various drugs on spontaneous contractile frequency of epididymal duct segments, we determined the contractile frequency in 2-minute-windows each - one immediately preceding the addition of a substance and the second starting 10 s after its addition.

Frequencies before and after addition of the different substances were compared using a one-way ANOVA (Friedman Test for paired non-parametric data) followed by Dunn's multiple comparisons test (GraphPad Prism, version 6.02 for Windows, GraphPad Software, La Jolla, California, USA, www.graphpad.com). Adjusted p-values are indicated.

## Results

### Visualization of spontaneous contractions in proximal regions of the epididymal duct

To assess contractile activity of the epididymal duct, we studied freshly isolated duct segments by a time-lapse imaging approach revealing spontaneous contractions (Movie S1).

**Figure 1:**
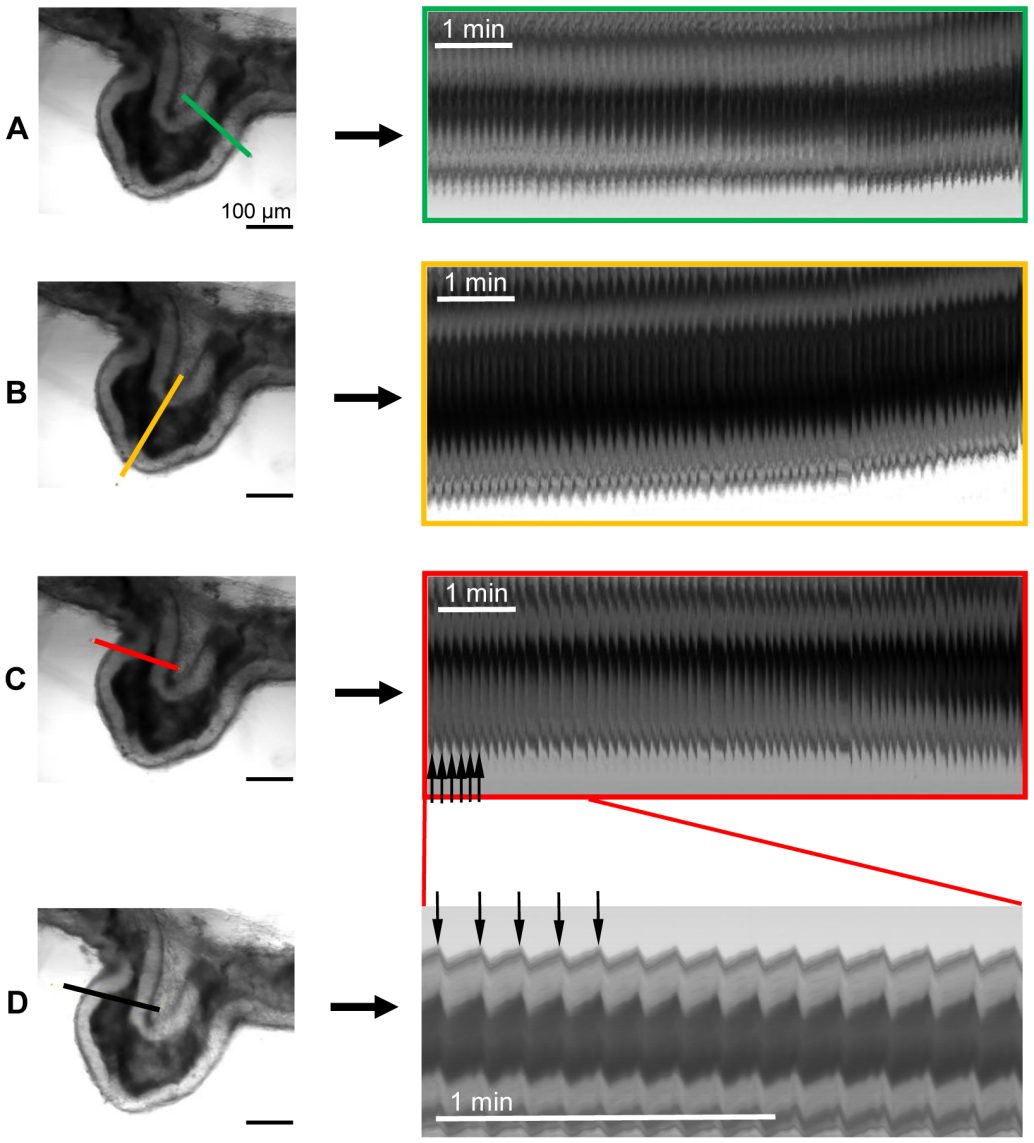
Simultaneous demonstration of contractile activity in different regions of one epididymal duct segment. Left side: Snapshot of the Movie S1 resulting from time-lapse digital photography of a piece of the epididymal duct in the caput region (scale bar: 100 μm). The different positions at which this time stack was virtually dissected are indicated by colored bars (A: green, B: yellow, C: red, D: black). Right side: Virtual sections through the time stack at indicated positions. Each contraction elicits a small movement of the epididymal duct with changes of its diameter resulting in a series of spikes (marked by vertical arrows). A–C: Virtual sections at different positions (1 frame/s). With the contractions spreading over the observed duct segment, the detection of contractions at different places yields equivalent results. D: Demonstration of contraction-derived pattern of spikes resulting from a movie captured at an accelerated rate of 7 frames/s providing more details. Contractile frequency in A–D is 7.5 beats/min.

Tracking of epididymal wall movements of this duct segment at a virtual section through the acquired image stack is possible and each wall movement can be depicted as a spike in the visualization overview at 1 frame/s (Fig. 1A–C, vertical arrows). Contraction frequency, described as the number of spikes per time, can quickly be assessed by counting. Accelerated movie capture (7 frames/s) provides a finer resolution in time (Fig. 1D), but frequencies can also be reliably assessed when the movies were captured at 1 frame/s (Fig. 1A–C). With the contractions encompassing the entire duct segment observed, the exact placement of the section through the time stack is of minor importance - this is illustrated by three cross sections (Fig. 1A–C, red, yellow and green outline, respectively) placed in different locations which yield the same contractile frequency.

**Figure 2:**
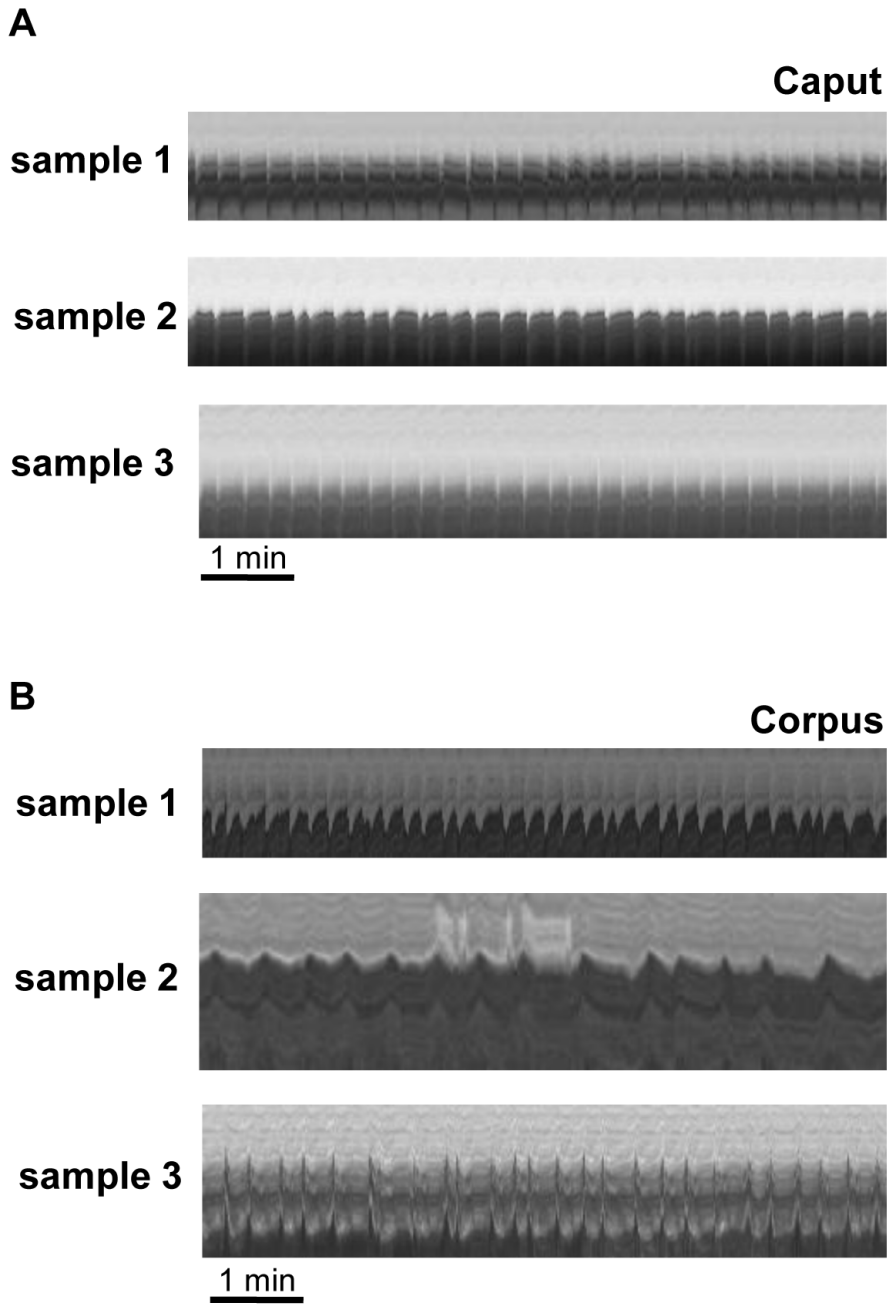
Spontaneous contractions in caput and corpus epididymidis. Visualization of contractility derived from virtual sections through the corresponding time stacks (as indicated in Fig. 1) in examples from duct segments originating from caput (A) and corpus (B) epididymidis of different individuals (samples 1, 2 and 3). All movies were captured at 1 frame/s. Regular spontaneous contractility is visible in all samples of the caput and corpus region.

To investigate whether spontaneous and regular contractile activity is consistently detectable in caput and corpus regions of the epididymis as previously demonstrated for mid-cauda regions by organ bath studies [15], we systematically examined various duct segments from caput and corpus regions by time-lapse imaging. A spontaneous and quite regular contraction pattern was observed in the caput and corpus segments of the epididymal duct investigated (Fig. 2A–B). Notably, we observed quick phasic contractions, but no obvious tonic contractions. The frequency of spontaneous contractions monitored by time-lapse imaging was in a range between 1.5 and 15 bpm with a peak around 5–6 bpm, this resembles the contraction frequencies previously observed in organ bath studies using mid-cauda segments [15].

The tissue used in Movie S1 and Fig. 1 was fixed at its extremities and otherwise floating in medium. A comparable pattern of contractile activity was observed in duct segments when embedded in collagen (Figs. 2A and 2B) indicating that the collagen lattice did not interfere with contractile activity.

To investigate whether peristaltic waves travel along a given duct segment, we observed duct segments at finer time resolution. Movie S2 shows the same experiment as Movie S1, but was captured at a higher speed of 7 frames/s allowing us to illustrate peristaltic contractions in the epididymal duct. Phasic contractions originated in a particular region of the epididymal duct and these waves propagated in one direction (Movie S2, asterisks mark progression of the first wave). With the finer time resolution, the propagation of phasic contractions within the wall of the epididymal duct was easily visible (Movie S2).

### cGMP-dependent effects on spontaneous contractile activity

Contractile activity of the epididymal duct is susceptible to various drug effects and our time-lapse imaging approach provides a tool for screening acute drug effects *ex vivo*. Since cGMP-related signaling has been shown to reduce contractile frequency in organ bath studies using mid-cauda segments [15], we investigated whether caput and corpus regions were also susceptible to cGMP-related signaling. We investigated the effect of sGC activation through the NO donor SNP and the PDE5 inhibitor sildenafil interfering with cGMP breakdown. Substances were added in inverse order to isolate the effect of each substance and possible interactions.

**Figure 3:**
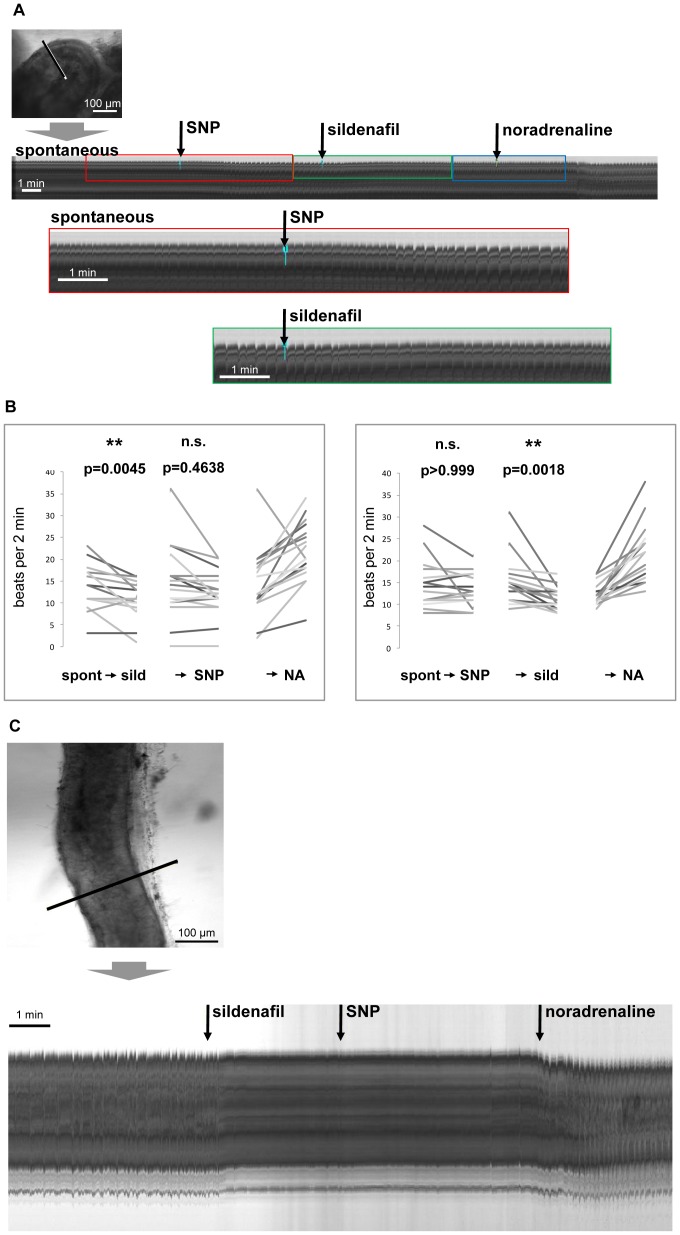
Visualization of sildenafil effects on spontaneous contractile activity. Effects of sildenafil and the NO donor SNP on spontaneous contractility of caput segments of the epididymal duct (A–C). A: Visualization of contractility derived from virtual sections through the corresponding time stacks (scale bar: 100 μm) in examples of SNP and subsequent sildenafil treatment of a duct segment from the caput region. Enlarging the regions that surround the time of drug addition, indicated by colored frames, illustrate transient effects of the substances. B: Statistical analyses compared the contractile frequency during 2 minutes preceding and following the addition of the substances. A non-parametric one-way ANOVA for repeated measurements (Friedman's test for paired samples) was used followed by Dunn's test for multiple comparisons. Adjusted p-values for each comparison are given in the graphs with “*” indicating p<0.05 and “**” indicating p<0.01. Statistical analyses of SNP and sildenafil treatments show that sildenafil significantly reduced contractile activity whether given alone or after SNP. In contrast, SNP effects remained non significant. “Spont” indicates spontaneous contractile frequency. C: Visualization of sildenafil and SNP effects in another duct segment originating from caput (scale bar: 100 μm). In this example sildenafil results in a complete loss of contractility. When the NO donor SNP was added in this situation, it was without additional effect as expected. The addition of noradrenaline at the end of the experiment lead to a resumption of contractile activity indicating that the duct segment was still viable. Movies were captured at 1 frame/s.

Sildenafil slowed down the frequency of spontaneous contractions (Fig. 3A–B) in caput and corpus of the epididymal duct or even abolished them (Fig. 3C, Movie S3). Sildenafil was also effective in epididymal duct segments pretreated with the NO donor SNP (Fig. 3A–B). In each case, the sildenafil effect was significant. Even though the addition of SNP seemed to elicit a small decrease in contractile frequency when given before or after sildenafil, the effect of SNP failed to reach significance (Fig. 3B). Noradrenaline was added at the end of the experiments mainly to prove viability of the tissue, and its addition obviously increased contractile frequency in our experiments.

**Figure 4:**
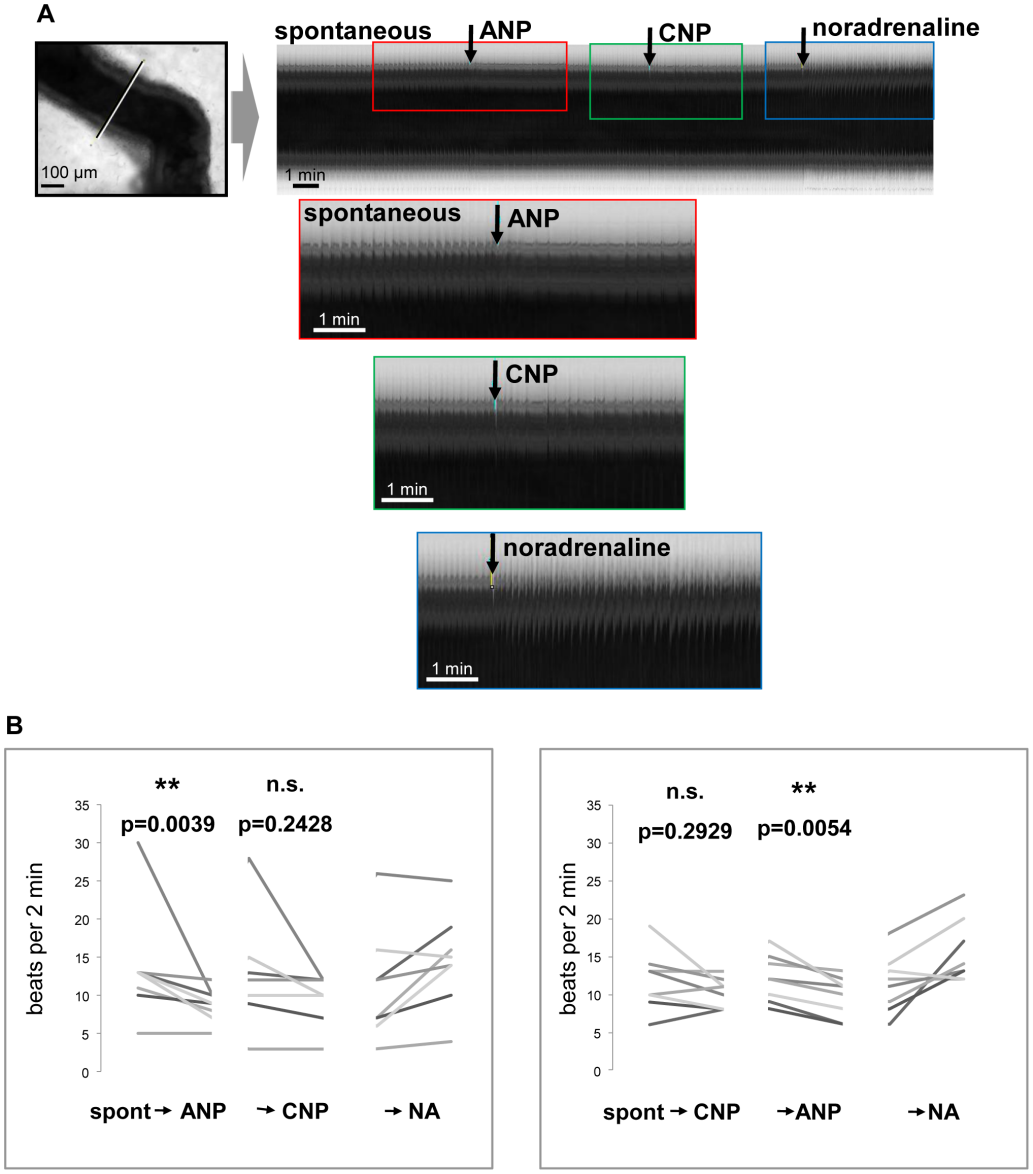
Visualization of ANP effects on spontaneous contractile activity. Effects of ANP and CNP on spontaneous contractility of corpus segments of the epididymal duct. A: Visualization of contractility derived from virtual sections through the corresponding time stacks in examples of ANP and subsequent CNP treatment of a duct segment from the corpus region (scale bar: 100 μm). Enlarging the regions that surround the time of drug addition, indicated by colored frames, illustrate transient effects of the substances. B: Statistical analyses compare the contractile frequency during 2 minutes preceding and following the addition of ANP and CNP. A non-parametric one-way ANOVA for repeated measurements (Friedman's test for paired samples) was used followed by Dunn's test for multiple comparisons. Adjusted p-values for each comparison are given in the graphs with “*” indicating p<0.05 and “**” indicating p<0.01. ANP significantly decreased spontaneous contractile activity and had a significant effect when given after CNP. CNP effects were non significant. “Spont” indicates spontaneous contractile frequency. Movies were captured at 1 frame/s.

The natriuretic peptides ANP and CNP increase cGMP generation through binding to their respective particulate receptors GC-A and GC-B. To evaluate effects of ANP and CNP and possible interactions, ANP was followed by CNP addition and vice versa. ANP significantly decreased contractile frequency in caput and corpus segments, regardless of whether ANP was applied before (Fig. 4A–B) or after CNP (Fig. 4B). Interestingly, the CNP effect seemed to be less pronounced and remained non-significant when given before or after ANP (Fig. 4A–B).

### Expression of the guanylyl cyclases GC-A, GC-B and sGC in the smooth muscle layer of the epididymal duct

**Figure 5:**
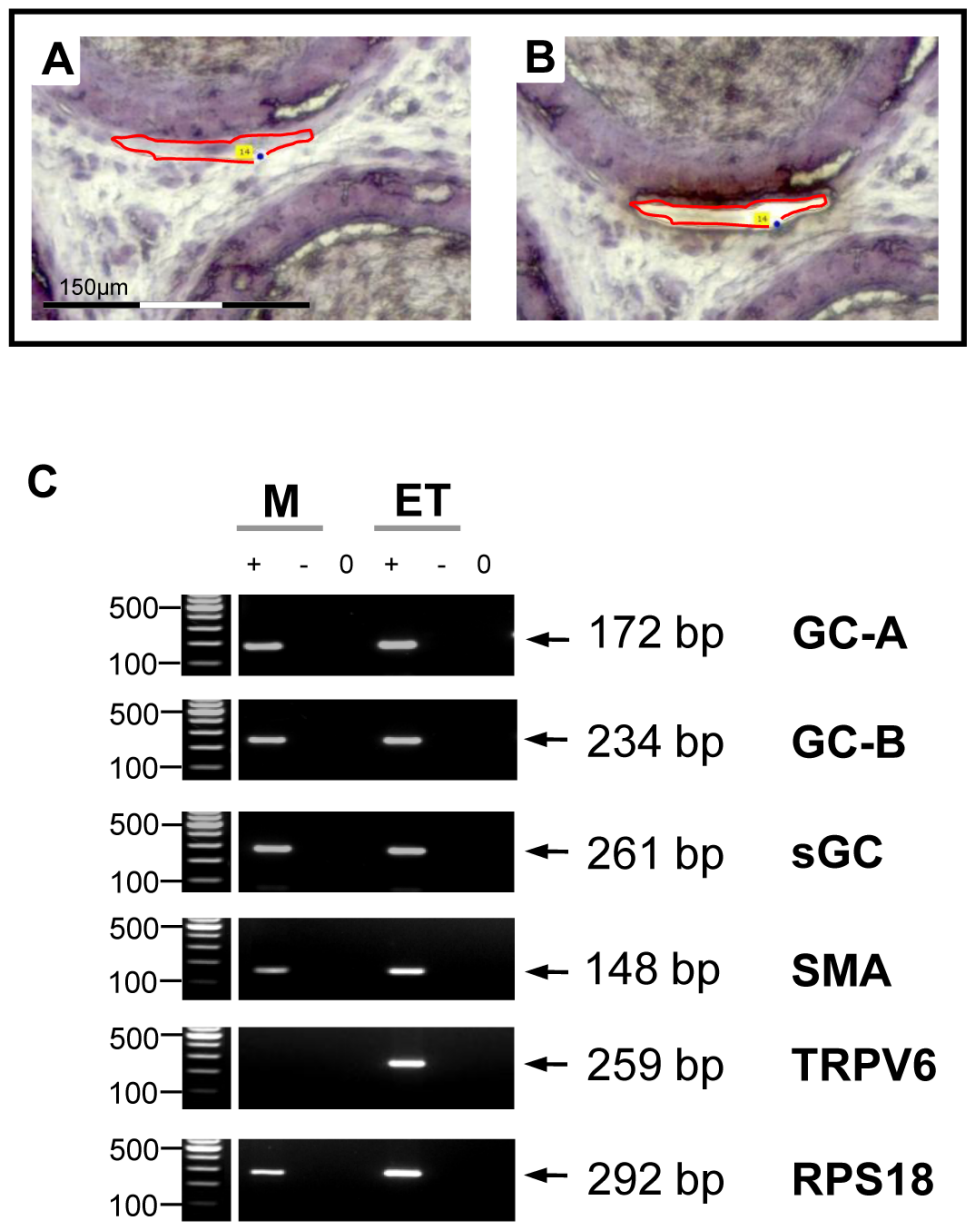
Demonstration of GC-A, GC-B and sGC expression in smooth muscle cells of the rat epididymal duct by LCM+RT-PCR. Laser-assisted microdissection (A,B) combined with RT-PCR (C) was used to localize GC-A, GC-B and sGC mRNA within the epididymis. A,B: Examples of excised segments of the epididymal smooth muscle layer. Scale bar corresponds to 150 μm. C: Microdissected epididymal smooth muscle cells (M) were analyzed by RT-PCR. Preparation from whole epididymis tissue (ET) served as positive control. The ribosomal protein RPS18 served as loading control. “+”, “−” and “0” indicate lanes with reverse transcriptase, without reverse transcriptase and water control, respectively. GC-A (172 bp), GC-B (234 bp) and sGC (261 bp) transcripts could be detected in microdissected smooth muscle layer. The quality of microdissection was assessed using additional primers for SMA (148 bp) as marker for smooth muscle cells and TRPV6 (259 bp) as marker for epithelial cells, respectively, to exclude the contamination of dissected parts of the smooth muscle layer with epithelial cells.

To confirm that components of the cGMP signaling system the functional activity of which was shown above are present in the rat within the smooth muscle layer of the epididymal duct, we performed laser-assisted microdissection combined with RT-PCR. Transcripts of the cGMP-generating enzymes (GC-A, GC-B and sGC) could be demonstrated in total rat epididymal tissue by RT-PCR (Fig. 5). To precisely localize these signaling components to the smooth muscle cell layer, we used laser-assisted microdissection to isolate the smooth muscle cell layer from the epididymal duct of rat epididymis sections, and subsequently analyzed the excised tissue by RT-PCR. All components of cGMP-generation were demonstrated by RT-PCR in the smooth muscle layer of the epididymal duct (Fig. 5). TRPV6 was used as a marker for epididymal epithelial cells [21] and its absence in the smooth muscle layer confirmed that no cross contamination with adjacent epithelial cells occurred during laser microdissection allowing to precisely ascribe GC-A, GC-B and sGC expression to the smooth muscle cell layer. Previous studies, in addition, have already revealed the presence of the cGMP-degrading PDE5 in smooth muscle cells of the rat epididymal duct [15].

### Visualization of luminal transport

**Figure 6:**
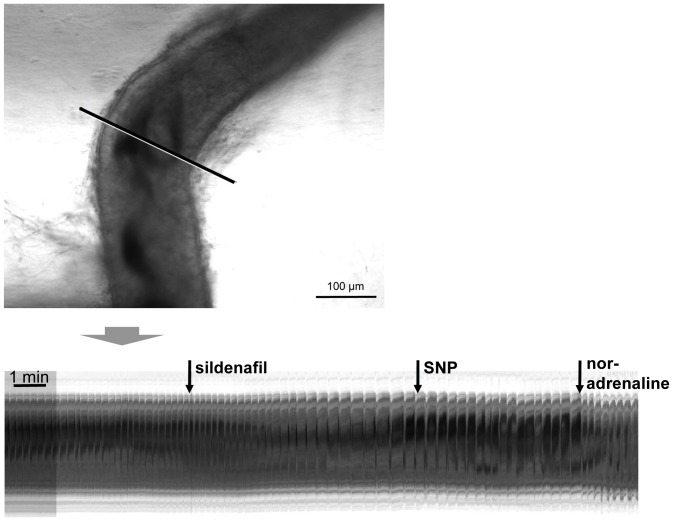
Visualization of transport of intraluminal contents. Snapshot of Movie S4 resulting from time-lapse imaging (1 frame/s) of a piece of the epididymal duct in the caput region (scale bar: 100 μm) showing net transport of intraluminal contents. In the cross section of the time stack, this net transport is indirectly visible when observing the pattern between the epididymis walls. The darker areas in the cross section do not remain constant over time indicating that intraluminal contents have moved out of the cross section.

Interestingly, in some duct segments effects of SNP and especially sildenafil on contractile pattern were barely discernible, whereas noradrenaline strongly accelerated contractile frequency (Movie S4, Fig. 6). Different to most of the other duct segments investigated (see Movie S1, S2, S3), these duct segments were additionally characterized by net transport of luminal content (Movie S4), in summary suggesting the existence of specified regions of the epididymal duct. In the cross section of the time stack in Fig. 6 (corresponding to Movie S4), this net transport was indirectly visible when observing the area between the walls of the duct: the darker areas in the cross section change over time indicating that intraluminal contents have moved out of the cross section. Although the analysis of the cross sections may hint to a movement of intraluminal contents, obviously this information can only be assessed reliably in the actual movie (Movie S4). As seen in this movie, noradrenaline seemed to additionally accelerate net transport of intraluminal contents. In the majority of other duct segments investigated, contractions also elicited movement of intraluminal contents, but mostly with a bidirectional flow towards the adjacent parts of the duct which did not contract at this moment. Frequently, an oscillating or pendular flow movement was visible (see especially Movie S3, prior to addition of drugs).

## Discussion

This manuscript describes the use of a time-lapse imaging-based approach to investigate contractile function of the epididymal duct in proximal regions of the organ and its susceptibility to cGMP-related signaling. Embedding duct segments in a soft collagen lattice stabilizes the tissue and enabled us to obtain stable virtual sections through the acquired image stack. This allowed rapid and reliable frequency analyses.

We illustrated contractile frequency of the epididymal duct by placing a virtual section through the image stacks and followed the movement of the walls over time. With each contraction translating into a small spike in the resulting overview, frequency and contraction pattern can be counted quickly. Since the contractions occur rather slowly, changes in frequency are subtle and barely detectable by simple observation. Time-lapse imaging, however, allows us to replay events in an accelerated fashion and - combined with the time stack analysis - reveals the contractile activity occurring during prolonged periods of time. In comparison to former studies describing contractility of the epididymal duct in rat by morphological studies [3], the data obtained with this method can be used for further statistical analyses much easier.

In addition, our approach allowed the simultaneous determination of contractile frequencies at different places of one individual duct segment. This was neither possible by previously used visual studies [3], [24] nor by pure physiological settings [15], [16], [25], [26] and it was interesting to see that the frequency of spontaneous contractions remained stable over the length of one isolated part of the epididymal duct.

As suggested before for different species [16], [24], [25], [27], [28] we observed a regular and phasic contractile activity in duct segments of all parts investigated in the caput and corpus regions. Direct information on contractile force, however, cannot be deduced directly by our experiments. The amplitude of the contractions of the epididymal wall or the inner diameter of the tubule may include some indirect information about contractile force. Within the assays, the observed amplitude of the epididymal wall contractions remained largely constant even after addition of the various substances that we screened.

Our time-lapse imaging approach allowed us to monitor the flow of intraluminal contents induced by wall contractions. In most cases, a pendular flow was generated by phasic contractions. Intraluminal contents moved bidirectionally towards the adjacent regions. We rarely observed net transport of intraluminal contents following vigorous contractions which corresponds to previous reports from Jaakkola et al. describing slow and fast flow patterns with clear reflux or net translocation of intraluminal contents, respectively [29], [30]. It is thinkable that contractile activity that really serves the propulsion of spermatozoa towards the the cauda epididymidis may be confined to particular regions of the epididymal duct and therefore be only occasionally observed, i.e. when these areas were actually sampled for the experiment. This suggests a functional compartmentalization of the epididymal duct with areas responsible for bringing spermatozoa in contact with the epithelium and others responsible for net transportation of intraluminal contents. Our observation that segments transporting intraluminal contents over some distance did not respond to sildenafil may hint towards a differential susceptibility of duct regions for cGMP-related signaling. However, this effect could not be isolated statistically due to the small number of observations. The idea that differential function and regulation is found in the different epididymal duct regions [31] warrants future systematic evaluation.

Our approach provides a reasonable tool to systematically test the effects of a variety of drugs and possible interactions on epididymal contractile frequency. Components of the cGMP signaling system, such as the ANP receptor GC-A, the CNP receptor GC-B and the NO-receptor sGC, have previously been described in the epididymis at the protein level of turtle, calf and rat [14], [32], [33]. Upon stimulation with the respective ligand, cGMP generation was observed [33]. Our RT-PCR data of laser-dissected smooth muscle cells from the rat epididymis complement these findings and add further localization information allowing to ascribe GC-A and GC-B to a smooth muscle cell localization. In addition, our time-lapse imaging data show a transient reduction of contractile frequency in response to the GC-A ligand ANP and a more sustained effect in mid-cauda segments [15] and support a functional role of natriuretic peptide signaling in the regulation of epididymal duct contractility in all regions of the epididymis and therefore a contribution to spermatozoa maturation. CNP effects in our experiments failed to reach significance. This finding corresponds to slight CNP effects described for the epididymal duct of the bovine caput [16]. In view of high CNP concentrations in the epididymis [34] and expression of the CNP receptor GC-B also in epithelial cells (in addition to smooth muscle cells) as well as CNP degradation by the apically localized epithelial neutral endopeptidase [35], this peptide may play a role in the regulation of epithelial function and fluid balance.

Comparable to CNP, the NO donor SNP also showed only slight effects in proximal regions of the rat epididymal duct. Again, this is in agreement with the bovine caput epididymidis [16]. Expression and activity of NO synthases, however, were shown in all parts of the epididymis [16], [36], [37] and the NO receptor sGC is localized in the smooth muscle cell layer. As yet clear NO-dependent effects on contractions of the epididymal duct were only found in distal regions of the rat [12], [15] and calf [16]. The differential susceptibility of distal segments towards NO-cGMP signaling as compared with proximal segments could be a function of the increasing thickness of the smooth muscle cell layer in cauda segments which makes subtle SNP effects visible more easily [7] or may relate to different, yet unknown, NO functions in the caput epididymidis. Whereas in caput and corpus regions contractile activity is needed to propel spermatozoa through the epididymis, the cauda region serves as a reservoir for spermatozoa and may need more mechanisms of relaxation to accommodate an increasing volume of spermatozoa. An increasing expression of sGC towards the distal regions (Müller, unpublished results) would contribute to this function.

PDE5 which limits cGMP signals was previously localized to the epididymal smooth muscle layer of all regions (caput, corpus, cauda) in human and rat tissue and shown to decrease contractile frequency in rat cauda epididymidis [15]. Our current investigation confirms an involvement of PDE5 also in the regulation of duct contractions of caput and corpus regions. The specific PDE5 inhibitor sildenafil which is increasingly used to treat erectile dysfunction [38] and pulmonary hypertension [39] may therefore have as yet unappreciated effects or side effects on epididymal function [15]. In isolated rat epididymal duct segments sildenafil rapidly lowered contractile frequency, but chronic sildenafil treatment of rats in vivo did not alter the regular contractile pattern of the epididymal duct. An additional role for other PDEs was suggested, but studies assessing their role in epididymal contractility are still needed.

In contrast to complicated cinematographic approaches used in the past, today modern digital image analyses are readily available. Our time-lapse imaging approach offers the possibility to systematically investigate the effects of various signaling systems on epididymal duct contractility.

## Supporting Information

Movie S1 **Contractile activity of the epididymal duct - spontaneous contractions.**
(AVI)

Movie S2 **Contractile activity of the epididymal duct, spontaneous contractions captured at a higher speed of 7 frames/s, illustrating peristaltic contractions in the epididymal duct.**
(AVI)

Movie S3 **Sildenafil abolish spontaneous contractions.**
(AVI)

Movie S4 **Effects of SNP and especially sildenafil on contractile pattern were barely discernible, whereas noradrenaline strongly accelerated contractile frequency.**
(AVI)
